# Job satisfaction and burnout of psychiatric nurses during the COVID-19 pandemic in China—the moderation of family support

**DOI:** 10.3389/fpsyg.2022.1006518

**Published:** 2022-09-08

**Authors:** Rui Jin

**Affiliations:** School of Government, Nanjing University, Nanjing, China

**Keywords:** psychiatric nurse, job satisfaction, job burnout, family support, COVID-19

## Abstract

**Purpose:**

The study aimed at investigating the state of psychiatric nurses’ job satisfaction, job burnout, and the moderating effect of family support between them in China during the COVID-19 pandemic.

**Materials and methods:**

Online self-report questionnaires were distributed and 212 psychiatric nurses participated in the research. Pearson correlation analysis, multiple stepwise regression analysis, and simple slope test were used for data analysis.

**Results:**

The results showed that the status of their job satisfaction (53.67 ± 10.72) and burnout (33.62 ± 13.84) did not reach a satisfactory level and job satisfaction had a significant negative impact on job burnout. Meanwhile, family support played a negative moderating role between the two variables.

**Conclusion:**

Psychiatric nurses suffered from job burnout in China during the COVID-19. Family support could have a counterproductive effect when the nurses were experiencing decreasing job satisfaction. It gave suggestions to the medical institutions and the government to improve the psychological well-being of the psychiatric nurses and even of all the medical staff.

## Introduction

Since the outbreak of the COVID-19 pandemic, healthcare workers have withstood much pressure and mental health burdens, and 80 percent of them stated that they suffered from a high level of perceived stress during the epidemic, which could reduce their job satisfaction (Preti et al., 2020; Lee et al., 2021). Moreover, psychiatric nurses may face more difficulties and tension in such situations (Lim et al., 2022). Being in the psychiatry unit, the nurses usually bear psychological distress and are frequently exposed to workplace violence (Hamaideh, 2012), and hence, the risk of job burnout occurs when they are always dealing with negative emotions like anger, pity, fear, etc. (van Dusseldorp et al., 2011). In China, hospitals have now adopted stricter regulations and more cumbersome workflows due to the COVID-19 pandemic. Some nurses were dispatched to aid in hard-hit cities and then were away from their homes and family. Under this condition, their work pressure would increase further and feel more distressful (Lim et al., 2022). Therefore, under this circumstance, job satisfaction and burnout of psychiatric nurses deserve our attention.

The concept of job satisfaction originated from the Hawthorne experiment by Mayo and George Elton, and its definition was proposed by Hoppock (1935). He defined job satisfaction as employees’ feelings with environmental factors in both psychological and physical aspects, that is, the subjective response of employees to the work situation (Hoppock, 1935). Many scholars have done related research on job satisfaction, such as factors affecting job satisfaction in different industries, the impact of job satisfaction on job performance, etc. (Spector, 1985; Judge et al., 2001). Nurses’ job satisfaction has a certain impact on nurses, patients, and medical units. Obtaining a sense of satisfaction and well-being from work can increase the overall life satisfaction of nurses and stimulate their initiative in providing efficient and high-quality health care services (Wu et al., 2006). After the outbreak of the COVID-19, the working demand of nurses have increased, which could lead to their dissatisfaction with their job and management of the hospital (Hering et al., 2022; Kim, 2022). Simultaneously, the nurses may feel fearful of the threaten of the pandemic, and this will influence their work performance and job satisfaction (Labrague and de Los Santos, 2021).

Job burnout was first proposed by American clinical psychologist Freudenberger in 1974 and was applied in mental health research. Since then, many scholars have conducted in-depth studies on this concept. Among those concepts, the most widely used is the definition of Maslach and Jackson. They defined job burnout as a combination of emotional exhaustion, depersonalization, and low personal accomplishments, which could often occur to people engaged in interpersonal work (Maslach and Jackson, 1981). During pandemic, both environmental-related and individual factors can lead to job burnout of medical staff (Khosravi et al., 2021). Environmental-related factors consist of role conflict, role ambiguity, and work overload, etc., while individual-related factors comprise loss of work-life balance and single status. Meanwhile, the hobbies can help the medical staff manage their emotions and stress at work (Salvagioni et al., 2017; Hu et al., 2020; Sasangohar et al., 2020). Nurses, as a high-risk group of job burnout, are paid attention by more and more scholars. The level of job burnout, the relationship between other factors (including job satisfaction) and burnout, and many other studies from different aspects have been done in the past few years (Happell et al., 2003; Hanrahan et al., 2010). Existing literature shows that job satisfaction has a significant negative impact on job burnout. The higher the employee’s job satisfaction is, the lower the burnout is. There is also much research examining the influence mechanism between the two factors (Dolan, 1987; Elit et al., 2004; Kluger et al., 2015).

Nowadays, the relationship between work and family has always been a concern. With the development of society, the relationship of “work-family facilitation” has gradually become a trend. Work-family facilitation refers to employees’ experiences in one role that will enhance their performance in another role, and work-family support is a specific form of work-family facilitation. This is a two-way concept, that is, supports the employees receive from work and family that are all beneficial to their lives (Li and Zhao, 2009). Some researchers have also studied the impact that family support generates on job satisfaction and job burnout. Karatepe and Kilic (2007) proposed that the support from spouses is significantly positively correlated with some outcome variables at work (such as working achievements and health status, etc.), and family support can help improve the employee’s physical health as well as job satisfaction (Labrague and de Los Santos, 2021). Lin (2014) found that family support could significantly reduce the burnout of medical staff. There are also some studies exploring the moderating effect of family support (Gao et al., 2020; Li et al., 2020). The COVID-19 could cause a dysfunctional level of anxiety among nurses. Under this circumstance, social support from the organization and family would help relieve this kind of anxiety (Labrague and De los Santos, 2020). The level of family support they perceived is positively correlated with their mental well-being (Ersin et al., 2021).

During the pandemic, we can often see news like this in China: a doctor or a nurse is sent to another city to help fight the epidemic while their young children are still staying in their hometown, and as a result, they cannot see each other for quite a long time. This phenomenon is so common that it reminded us to consider whether this will cause work-family conflict and then impact job satisfaction and burnout of medical staff. However, existing studies have not tested the level and the interaction of these two factors in the context of psychiatric medical institutions during the epidemic in China, nor have they investigated the role of family support for medical staff.

Based on the above, the study will shed light on the current state of job satisfaction and burnout of Chinese psychiatric nurses and the relationship between the two factors. Afterward, the moderating role of family support will be checked as well.

## Materials and methods

### Subjects and data collection

This research is a cross-sectional descriptive study conducted with a self-reported questionnaire.

The subjects of the study are in-service psychiatric nurses who have obtained vocational qualification certificates from four Grade-A tertiary hospitals in Jiangsu Province, China. All these nurses have worked in psychiatric units for more than 2 years and they totally understood the goal of this research. Simple random sampling was adopted in this study. All the subjects worked at the frontline and worked night shift every 3 days. They needed to undertake different kinds of extra tasks related to the pandemic, such as temperature measurement and registration for every visitor to their hospital. The nurses rehired after retirement were excluded because they might be responsible for some relatively easier assignments. From November to December 2021, a total of 229 questionnaires were distributed and 212 valid questionnaires were retrieved, with an effective rate of 92.58%. Among the subjects, 190 were women, accounting for 89.62%. 65.09% of the subjects had a bachelor’s degree or above and 76.42% of them were married. Only 27.36% of the nurses had not reached a senior level and a total of 65.09% had worked in psychiatric units for more than 5 years. The other demographic information of respondents is as below (Table 1).

**TABLE 1:** Demographic information of respondents.

Items	Category	Frequency	Ratio
Gender	Male	22	10.38%
	Female	190	89.62%
Age	Below 25	34	16.04%
	25–29	72	33.96%
	30–29	79	37.26%
	Above 40	27	12.74%
Education	Diploma	64	30.19%
	Bachelor	137	64.62%
	Master or higher	1	0.47%
	Others	10	4.72%
Job title	Nurse	58	27.36%
	Senior nurse	75	35.38%
	Nurse-in-charge	65	30.66%
	Associate chief nurse	14	6.60%
Marriage	Married	162	76.42%
	Unmarried	48	22.64%
	Divorced	2	0.94%
Years of working	2–3	27	12.74%
	3–5	47	22.17%
	6–9	59	27.83%
	Above 10	79	37.26%

### Instruments

#### Job satisfaction scale

The job satisfaction scale designed by Warr et al. (1979) has been widely used in nursing management research internationally. I adopted its Chinese version which was designed by Hong (2007) in order to get precise results in the context of Chinese society. The scale includes two dimensions: status at work and interpersonal relationships at work, with a total of 15 measurement items. I used a five-point Likert scale ranging from one to five, which means strongly dissatisfied to strongly satisfied. The items are shown in Table 2.

**TABLE 2:** Measurement items of job satisfaction.

Dimensions	Measurement items
Status at work	Variation of job content
	Management of the hospital
	Payment
	Working hours
	Attention given to suggestions
	Freedom of working style
	Stability of job
	Opportunity to make use of abilities
	Opportunity of promotion
	Working conditions
	Responsibilities
Interpersonal relationships at work	Direct leader
	Colleagues
	Relationship between leaders and subordinates
	Recognition of good performance

In this study, the Cronbach’s α of the overall scale, “status at work” dimension, and “interpersonal relationship at work” dimension was 0.969, 0.965, and 0.916, respectively, which indicated good reliability.

#### Job burnout scale

I used the MBI-GS job burnout scale (Chinese version). It was modified by Chao-Ping and Shi-kan (2003), including three dimensions: emotional exhaustion (five items), depersonalization (four items), and low personal accomplishment (six items). Each item was measured using a seven-point Likert scoring scale (zero “not at all” to six “always”). The higher the score is, the more serious the job burnout is.

The Cronbach’s α of the overall scale, emotional exhaustion, depersonalization, and low personal accomplishment was 0.911, 0.956, 0.965, and 0.953, respectively.

#### Family support scale

King et al. (2010) developed scales for support from work and support from family and divided work-family support into four types, among which include official support, unofficial support, emotional support, and instrumental support. On the basis of their research, Chinese scholars Li and Zhao (2009) developed the “Family-Work Support Scale,” which consists of four dimensions: organizational support, leadership support, emotional support, and instrumental support. The first two are about social support while the latter two identify family support. Therefore, I adapted the dimension of emotional support (six items) and instrumental support (four items) to measure the family support of the subjects. Emotional support includes care, love, and encouragement whereas instrument support includes guidance, assistance, tangible support, and problem-solving actions. I used a seven-point Likert scale ranging from one to seven, which indicates strongly disagree to strongly agree (The items are shown in Table 3).

**TABLE 3:** Measurement items of family support.

Dimensions	Items
Emotional support	My family members always offer different opinions and views on my work.
	My family members can always understand me when I have trouble at work.
	My family members always share the difficulties of work with me.
	My family members always encourage me when I am tired of working.
	I always talk to my family members when I have problems at work.
	My family members always comfort me when I have problems at work.
Instrumental support	My family members always give me some personal space after work.
	My family members always do more housework when I am busy at work.
	I always feel comfortable when talking with my family members about work.
	My family members are interested in my work.

The Cronbach’s α of the overall scale, emotional support, and instrumental support were 0.943, 0.946, and 0.921, respectively.

### Methods of data analysis

Firstly, descriptive statistics of the retrieved data was performed to learn about the average score and the standard error of each variable and dimension. Secondly, Pearson correlation analysis was conducted to ensure discriminate validity of the dimensions. Thirdly, I did multiple stepwise regression to check the relationship between job satisfaction and burnout. Lastly, the moderation of family support was tested by constructing two regression models. The first model contained job satisfaction, family support, and job burnout, and in the second one the interaction term of job satisfaction and family support was added additionally. Moreover, simple slope test could help show the moderation more intuitively.

## Results

I used SPSS 22.0 and PROCESS for statistical analysis. The enumeration data was described by frequency and composition ratio while the measurement data was described by mean and standard deviation. Statistical analysis methods applied include Pearson correlation analysis, multiple stepwise regression analysis, simple slope test, etc.

### Descriptive statistics of job satisfaction and job burnout

The total score of psychiatric nurses’ job satisfaction was (53.67 ± 10.72), and the average score was (3.66 ± 0.70). According to the standard, the overall job satisfaction has not yet reached a satisfactory level, and the score of “status at work” is lower than that of “interpersonal relationships at work.” In addition, psychiatric nurses had mild job burnout as a whole. The total score of job burnout was (33.62 ± 13.84), and the average score was (2.17 ± 0.96). The score of “low personal accomplishment” was the highest. Meanwhile, according to statistics, 26.07% of subjects are in a state of severe job burnout. Overall speaking, the nurses in the sample had relatively high mean levels in job burnout and its two subscales, and reported low to moderate levels in job satisfaction and its two dimensions. The results are displayed in Table 4.

**TABLE 4:** Descriptive statistics.

Variables	Total score	Dimensions	Total score of each dimension	Average score of each dimension
Job satisfaction (15 items, 1–5 points)	53.67 ± 10.72	Status at work	38.30 ± 8.31	3.48 ± 0.76
		Interpersonal relationships at work	15.37 ± 2.83	3.84 ± 0.71
Job burnout (15 items, 0–6 points)	33.62 ± 13.84	Emotional exhaustion	10.67 ± 5.68	2.13 ± 1.34
		Depersonalization	6.67 ± 5.64	1.67 ± 1.41
		Low personal accomplishment	16.27 ± 8.37	2.71 ± 1.40

### Pearson correlation analysis

Pearson correlation analysis was conducted to test the discriminate validity of the data. The Pearson correlation coefficients between the variables are listed in Table 5. The two dimensions of job satisfaction and the two dimensions of family support were significantly negatively correlated with job burnout. Apart from this, dimensions of family support were significantly positively correlated with the dimensions of job satisfaction. We could conclude that the discriminate validity of the samples is acceptable because the diagonal elements are significantly larger than the correlation of a certain dimension with any of the other dimensions and all values are above 0.5.

**TABLE 5:** Test of discriminate validity.

	1	2	3	4	5
1 Status at work	0.865				
2 Interpersonal relationships at work	0.805**	0.894			
3 Emotional support	0.542**	0.507**	0.894		
4 Instrument support	0.496**	0.466**	0.663**	0.900	
5 Job burnout	−0.592**	−0.554**	−0.489**	−0.363**	0.872

*P < 0.05, **P < 0.01, and ***P < 0.001.

### Impact of job satisfaction on job burnout

I used multiple stepwise regression to analyze the relationship between job satisfaction and job burnout. Taking the average score of “Status at work” and “Interpersonal relationships at work” as the independent variables, and the average score of job burnout as the dependent variable, I explored the relationship between job satisfaction and burnout. It was found that both independent variables entered the regression and interpreted 36.7% of the variation in job burnout. As the level of satisfaction with “Status at work” and “Interpersonal relationships at work” rise, the level of job burnout will decrease significantly. Meanwhile, the change of satisfaction with “Status at work” can account for more variation in job burnout than that of “Interpersonal relationships at work.” The result is as below (Table 6).

**TABLE 6:** Results of regression (*N* = 212).

Variables	Regression coefficient	Standardized error	Standardized regression coefficient	*t*-value	*P*-value
Status at work	−0.350***	0.078	−0.415	−4.469	0.000
Interpersonal relationships at work	−0.198*	0.084	−0.220	−2.371	0.019
Constant term	4.430***	0.194	−	22.793	0.000

*P < 0.05, **P < 0.01, and ***P < 0.001, R = 0.606, R^2^ = 0.367, F = 60.684, P = 0.000.

### Moderating effect of family support

**FIGURE 1:**
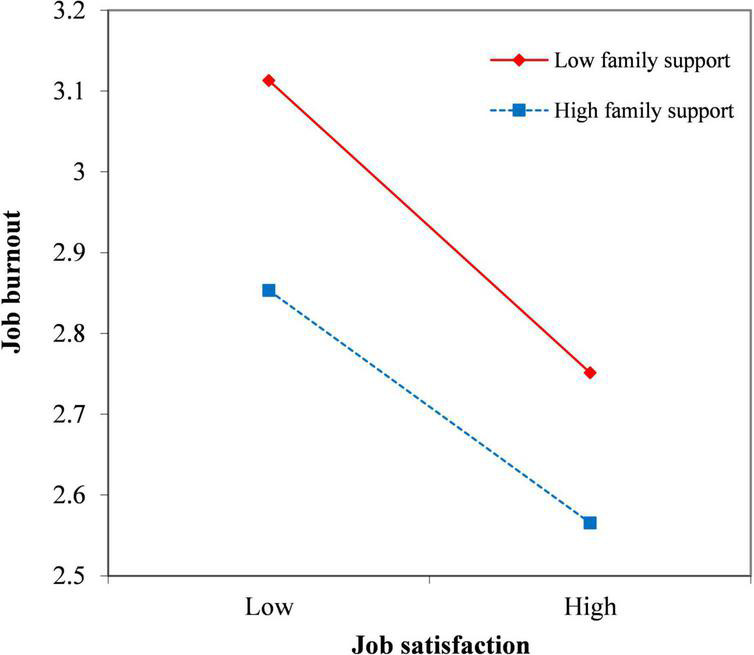
Moderation of family support.

Hierarchical regression analysis was adopted to examine the moderating effect of family support between job satisfaction and burnout. Firstly, in Model 1, I added job satisfaction and family support into the regression. It could interpret 37.8% of the variation of job burnout, and the regression coefficient of family support was −0.170, which showed that family support had a significant negative impact on job burnout (*P* < 0.05). Secondly, I furtherly added the interaction term of family support and job satisfaction to Model 2. The interpretation of job burnout reached 38.7% (*P* < 0.001) and the Δ*R*^2^ was 0.9%. The regression coefficient of the interaction term was 0.767 (*P* < 0.05). It revealed that family support had a significant interfering moderating effect between job satisfaction and job burnout, that is, compared with those with high family support, the improvement of job satisfaction has a more significant influence on the decrease of job burnout of psychiatric nurses with low family support (Figure 1). In Figure 1, the *P*-value of slope for low family support was 0.046 and that for high family support was 0.133. In Model 2, family support also had a significant negative effect on job burnout (*P* < 0.01). The specific results are shown in Table 7.

**TABLE 7:** Moderating effect of family support.

	Independent variables	Standardized regression coefficient	*R* ^2^	Adjusted *R*^2^	*t*-value	*P*-value
Model 1	Job satisfaction	−0.506	0.384	0.378	−7.591	0.000
	Family support	−0.170			−2.547	0.012
Model 2	Job satisfaction	−0.911	0.395	0.387	−4.316	0.000
	Family support	−0.618			−2.672	0.008
	Job satisfaction × family support	0.767			2.023	0.044

In the following, I adopted three sub-dimensions of job burnout: emotional exhaustion, depersonalization, and low personal accomplishment as dependent variables, and used family support as the independent variable. Then I conducted a hierarchical regression analysis again to further explore the moderating effect of family support.

When the dependent variable is emotional exhaustion:

I put job satisfaction and family support in the model, the adjusted *R*^2^ was 0.449, which interpreted 44.9% of the variance in emotional exhaustion. After adding the interaction term of job satisfaction and family support, *R*^2^ increased by 0.9% (*P* < 0.001), and the standardized regression coefficient of the interaction item was 0.750 (*P* < 0.05), indicating that family support had a significant interference in moderating effect on job satisfaction and emotional exhaustion, that is, for psychiatric nurses with low family support, the improvement of job satisfaction is more conducive to the relief of emotional exhaustion.

When the dependent variable is depersonalization and low personal accomplishment:

After putting the interaction term of job satisfaction and family support in the regression of job satisfaction, depersonalization, and family support, the adjusted *R*^2^ was 38.2% (*P* < 0.001), but the interaction term’s *t*-value was 1.847, *P* = 0.066 (>0.05), demonstrating that family support has no moderating effect between job satisfaction and depersonalization.

In the same way, I added the interaction term to the regression of job satisfaction, low personal accomplishment, and family support, the adjusted *R*^2^ was 0.3% (*p* > 0.05), and the *t*-value of the interaction term was 0.313, *P* = 0.755 (> 0.05), manifesting that family support has no moderating effect between job satisfaction and of low personal accomplishment psychiatric nurses.

## Discussion

### Job satisfaction and burnout of psychiatric nurses

The results showed that the total score of psychiatric nurses’ job satisfaction was (53.67 ± 10.72), and the total average score was (3.66 ± 0.70), indicating that it has not reached a satisfactory level, which is consistent with the result of research by Zhou et al. (2019). The score is much lower than that of psychiatric nurses in other countries (before the COVID-19) (Sharp, 2007; Zheng et al., 2017). Furthermore, 18.4% of the subjects’ job satisfaction was lower than 45 points, showing severe job dissatisfaction. The average score of interpersonal relationships at work (3.84 ± 0.71) was higher than that of status at work (3.48 ± 0.76), but neither of them reached a satisfactory level. It means the nurses had a relatively good relationship with colleagues and leaders and were more dissatisfied with the work itself. Among the dimension of “status at work,” three items with the lowest satisfaction scores were payment, opportunities for promotion, and the attention given to the suggestions, which may be related to psychiatric nurses’ high work pressure, huge workload, low salaries, and other welfare-related issues, as well as relevant to the lack of professional development opportunities (Walker, 2008). It is also possible that many of the subjects have worked for a long time and have not been promoted for many years due to various reasons, which has led to their increased job dissatisfaction (Malik et al., 2012; Razak et al., 2018).

In terms of job burnout, the overall score of the subjects in our study was (33.62 ± 13.84), which belonged to the level of mild job burnout. However, 26.07% of them had an average score of three points or more, which means they have serious job burnout, which may be related to factors such as their work environment and workplace violence (Imai et al., 2004; Seo et al., 2019). Of the three dimensions of job burnout, low personal accomplishment scored the lowest. It can be seen that psychiatric nurses tended to gradually reduce their personal accomplishments during the process of work, and even doubt their personal value. This might be relevant to the current status of the nursing profession, the relationship with patients, and the social support they have received (Van Bogaert et al., 2013; Woodhead et al., 2016). If burnout continues to aggravate, it will drive the nurses to leave their job (Chen et al., 2019).

During the epidemic, the diagnosis and treatment procedure in most Chinese hospitals have become increasingly complex, with a series of processes of registration, health code verification, nucleic acid testing, etc. Under this circumstance, the number of employees of medical institutions has not grown correspondingly, as a result of which most of these jobs are now performed by nurses. Especially in the psychiatry units, many patients are often unable to handle these must-do tasks by themselves, which has made the nurses’ work a lot more cumbersome and ultimately increase their job burnout.

### Relationship between job satisfaction and job burnout

Multiple regression analysis showed that the two dimensions of nurses’ job satisfaction—status at work (*P* < 0.001) and interpersonal relationships at work (*P* < 0.05)—both entered the regression equation. This indicated that these two dimensions were the influencing factors of psychiatric nurses’ job burnout, which could explain 36.7% of the variation of job burnout in total. And both the two had negative correlations with burnout, that is, the better the status and the interpersonal relationship at work, the lower the level of job burnout. In general, job satisfaction of psychiatric nurses had a negative correlation with their job burnout, which is consistent with existing studies (Liu et al., 2019; Payne et al., 2020).

### Moderating effect of family support

In this study, through hierarchical regression analysis, it was found that family support of psychiatric nurses had a significant negative moderating effect on the relationship between job satisfaction and job burnout. In other words, it would weaken the negative relationship between job satisfaction and burnout; the burnout of nurses with low family support would be more alleviated with the increase in job satisfaction. Further research revealed that family support mainly affected the emotional exhaustion dimension of job burnout, and had a negative moderating effect between job satisfaction and emotional exhaustion. This result seems to be contrary to our conventional knowledge.

Existing research shows that social support (including support from supervisors, friends, and family members) can reduce the feeling of burnout in nursing staff. Also, family support plays a positive moderating role between doctors’ emotional exhaustion and subjective well-being (Wang et al., 2020). But according to our research, family support does have a negative moderating effect, which means it may have a counterproductive effect when the nurses are feeling more and more dissatisfied with their job.

Conservation of Resources theory (COR) suggests that resources and demands affect strain within a person’s idiosyncratic ecology (Hobfoll, 2001). Individuals have a tendency to work hard to maintain, protect, and build what they perceive to be valuable resources, and the potential or actual loss of valuable resources can cause strain on the individual. In this context, psychiatric nurses with low family support, are more reliant on their job to obtain resources to meet their demands, and once their status at work and interpersonal relationships have improved, they will be more sensitive to this change and thereby gain more resources from work. In this way, the improvement of job satisfaction has a more significant impact on their job burnout. On the other hand, nurses with high family support, are more likely to get resources from the outside of their job (especially from family) and consequently are less vulnerable to changes at work. In this light, when situations at work go bad, family support can lead to unwanted outcomes that will worsen job burnout.

There are other possible explanations for this result. Kaufmann and Beehr (1986) proposed that too much family support will bring pressure on employees to some extent. When they are unable to fulfill their duties and obligations in the family due to their job, they will feel guilty about this, and the guilt can further evolve into negative emotions toward work, resulting in job burnout. In addition, according to Equity Theory, employees need to maintain a sense of distributional fairness. When psychiatric nurses receive high family support, they will correspondingly have higher expectations and requirements on their job, such as higher payment, promotion, etc. When these factors cannot meet their expectations, it will cause job burnout, especially emotional exhaustion.

### Implications of the study

The study revealed the job satisfaction and burnout of Chinese psychiatric nurses during the epidemic and drew conclusions about the impact of job satisfaction on job burnout. In specific, there occurred job dissatisfaction among psychiatric nurses and they had a certain degree of job burnout. Meanwhile, it looked into the influence path of job satisfaction on burnout and found the negative moderating effect of family support. Our research applied the theory of Conservation of Resources in the context of psychiatric nurses’ job burnout, confirming non-work resources could impact people’s strain experience significantly as well as enriching the application of the theory.

On the basis of the results, we can draw up some effective measures to relieve job burnout of psychiatric nurses during the COVID-19 pandemic. First, the hospitals can streamline their workflow and hire more staff to reduce the nurses’ workload and create a good work environment for them. During daily work, they ought to pay attention to the nurses’ mental state, and for nurses with a high level of job burnout, psychological intervention, and organizational adjustment should be taken when necessary to alleviate their strain. Second, the hospitals should especially care about the nurses’ personal accomplishments. Hospitals can give recognition to the outstanding employees from time to time and take their advice on work into account. Third, the medical institutions can form organizations such as a “hospital-family alliance,” from which they could listen and talk to the family of the employees, and thus know about their needs. In this way, the policy is able to be switched flexibly concerning the nurses’ physical and mental status. Fourth, studies have shown that personality traits such as high novelty-seeking and neuroticism have a positive correlation with the level of burnout, which indicates that the hospitals can take the nurses’ personalities into account in order to come up with targeted measures to relieve their job burnout (Khosravi et al., 2020; Khosravi, 2021).

### Research limitations

Firstly, one of the limitations of the study is that I could only distribute the questionnaires online instead of doing field surveys due to the strict policies. Therefore, not all the subjects were from the hard-hit areas and some of them had not cared for patients with COVID-19 infections, which might undermine the representativeness of the results. Secondly, the samples size is relatively small and we only had a few samples of male nurses. This has restricted us from analyzing the differences between male and female samples, which could be quite interesting. Thirdly, the study adopted self-reported questionnaire to collect data, which can lead to possible bias. The participants’ feelings and scoring criteria are subjective, and everybody can have their own response styles to the questions. Moreover, their answers may only reflect the feelings during the assessment and not reveal the real emotions they have suffered in daily work. Fourthly, the results of the research were on the basis of a cross-sectional design. Therefore, accurate predictions cannot be guaranteed between job satisfaction and burnout, and the results should be examined further in longitudinal studies.

## Conclusion

This research focused on job satisfaction and burnout of psychiatric nurses in China under the COVID-19 pandemic and looked into the moderating effect of family support. Questionnaires were designed and distributed and the results showed that the nurses were not that satisfied with their current work status and some of them had relatively severe job burnout. What’s more, the main contribution of this study is the finding of the paradoxical effect of family support. This study provided insight into the relationship between job satisfaction, burnout, and family support and applied the Conservation of Resources theory in the context of psychiatric nurses. Such an outcome could remind the medical institutions and the authorities to pay attention to the mental state of medical staff as well as improve their management.

## Data availability statement

The raw data supporting the conclusions of this article will be made available by the authors, without undue reservation.

## Ethics statement

Ethical review and approval was not required for the study on human participants in accordance with the local legislation and institutional requirements. The patients/participants provided their written informed consent to participate in this study.

## Author contributions

RJ conducted the survey, analyzed the data, wrote the manuscript, and approved the submitted version.
